# The Influence of Cognitive Load on Distractor-Response Bindings

**DOI:** 10.3389/fpsyg.2021.696353

**Published:** 2021-07-26

**Authors:** Tarini Singh, Torsten Schubert

**Affiliations:** Department of Experimental Psychology, Martin-Luther University Halle-Wittenberg, Halle, Germany

**Keywords:** distractor-response binding, stimulus-response binding, central attention, action control, cognitive load

## Abstract

Binding theories postulate an integration of stimulus and response features into temporary episodic traces or event files. In general, in the visual binding literature, attention is considered to be necessary to feature binding, and a higher cognitive load can lead to worse performance. On the other hand, in stimulus-response binding theories, central attention is not regarded as necessary in binding effects. A possible discrepancy between the visual feature binding findings and the findings in stimulus-response binding studies could lie in the amount of central load implemented, whereas another discrepancy was related to a specific type of process that was manipulated. In the present study, load was manipulated in three levels, such as no load, low load, and high load, and the binding effects were tested under each condition. Load was manipulated by using a secondary task, which was to be carried out simultaneously with the primary task. Additionally, the influence of targeting different working memory processes (maintenance and updating) was examined by varying the time point of the presentation of the secondary task. The results indicate that, under high load, binding effects are observed if memory contents are merely maintained, but not observed when memory contents are actively updated.

## Introduction

The world around us is filled with a number of different stimuli, which are more or less relevant to our current goals. All of these stimuli are made up of a number of different features such as color, shape, location, orientation, and others. In order to properly perceive these stimuli and process them according to our goals, we must integrate or bind these different features together to form a perceptual representation. Treisman (1988) called this a “binding problem,” and suggested that, in her feature integration theory (FIT) of attention, all of these different visual features must be integrated to accurately perceive objects around us. This feature binding is necessary not only in the perception of objects but also in the planning of any actions, for example, we must plan which effector is to be used, in which direction a movement is to be made, and what orientation the effector must be to achieve the goal. Thus, both perception and action require feature binding.

Hommel (1998) and Hommel et al. (2001) suggested that the features of stimuli and responses to them are automatically integrated or bound together and stored in temporary episodic traces called an *event file* (or similarly, *S-R episode*, Waszak et al., 2003 or *instances*, Logan, 1988). Hommel et al. (2001) suggested that perception and action planning are based on the same set of representational codes and event files contain bindings between the features that belong to the stimulus being perceived as well as the features of the action being planned toward that stimulus—*stimulus-response binding*. These bindings not only influence the current action and perception but also influence future actions. Repeating only some of the features of a previous event file, i.e., a so-called *partial repetition*, results in costs in terms of longer reaction times (RTs) or more errors as the retrieved event file does not completely match the new stimulus, and the retrieved event file thus causes interference. On the other hand, repeating all of the features or none of them do not result in costs as, in the former, the new stimulus is in complete match with the retrieved event file, and in the latter, as no features are repeated, no event file is retrieved. It must be noted here that, depending upon the task requirements, the behavioral pattern in the complete repetition and complete change conditions differs. If the task requires stimulus discrimination, then the performance is facilitated in the complete repetition condition as the repeated stimuli retrieve the accurate response (e.g., Forster and Davis, 1984; Henson et al., 2014). If, however, a simple detection of stimuli is required, then the performance is slowed in the complete repetition condition due to *inhibition of return* (Lupiáñez et al., 2013). The present discussion will focus on discrimination tasks.

Along with the features of stimuli that are relevant to our goals, even the features of irrelevant stimuli that occur in close temporal contiguity with the relevant stimulus are stored in the event files and bound to the response—*distractor-response binding* (e.g., Frings et al., 2007). Such distractor-response bindings can also influence future actions. A repeated distractor can retrieve the event file in which it is bound, which also includes the response information. The effect of a repeated distractor depends upon whether the response is repeated. If the distractor and the response are both repeated, then the repeated distractor facilitates responding. The repeated distractor retrieves the previous event file, which contains the response information that is still accurate as the response must be repeated. On the other hand, if the response is changed, then a repeated distractor will interfere with responding as the response information contained in the retrieved event file is no longer appropriate and the response must now be changed. The aim of the present study is to examine the effect of central attention, or cognitive load, on the strength of distractor-response binding effects.

### Binding and Central Attention

Johnston et al. (1995) differentiated input attention influencing a parallel processing of visual stimuli from central attention influencing higher mental functions (see also Reimer et al., 2015, Reimer and Schubert, 2019, 2020; however, see Tamber-Rosenau and Marois, 2016 for a hierarchical account). For the present purposes, it is important to differentiate between visual attention and central attention. Visual attention can be subdivided into spatial attention, i.e., focusing attention on a specific location, and feature-based attention, i.e., allocation of attention to specific features of objects, e.g., color, shape, etc. (Carrasco, 2011). Central attention, on the other hand, is critical to various higher mental processes (Johnston et al., 1995). Limitations of central attention resources or increased demand has been observed to affect a number of processes, e.g., human performance deteriorates when two tasks have to be performed in parallel as evidenced by a few studies on dual task performance (e.g., Pashler, 1984; Schubert, 1999), lower working memory capacity leading to weaker distractor inhibition (e.g., Conway et al., 1999) and more mind wandering (Schurer et al., 2020). Increasing demands on central attention, for instance, by increasing working memory load leads to a larger distractor interference (de Fockert et al., 2001; however, see Gil-Gómez de Liaño et al., 2016 for conflicting results), reduced Simon effect (Wühr and Biebl, 2011) and reduced post-conflict adaptation (Soutschek and Schubert, 2013; Soutschek et al., 2013). The influence of central attention on binding effects, however, is not as well-understood. Some theories consider binding to be independent of central attentional resources (e.g., Theory of event coding, Hommel et al., 2001), whereas other theories consider attention to be crucial to bindings (e.g., FIT of attention, Treisman, 1988).

According to the theory of event coding, the formation of event files is an automatic process that occurs when we perceive an object or plan an action, and is largely unaffected by central attention capacity. Indeed, previous studies have provided evidence that bindings or event files are created even under increased attentional requirements. For instance, Hommel (2005) instructed participants to perform a secondary auditory go/no-go task or an auditory discrimination task in parallel with a visual identification task. He observed that even when performing the secondary task, the integration of stimulus and response features was not affected. Based on these results, it could be argued that the integration of stimulus and response features is independent of any attentional resources. However, the primary and secondary tasks in that study were presented in two different modalities, which might offer an alternative explanation (however, see Morey et al., 2011; Wahn and König, 2017, for shared attentional resources). In another study of examining bindings under increased attentional requirements, Moeller and Frings (2015) specifically examined the effect of central attention on distractor-response bindings within a single modality. They observed no reduction in binding effects due to their secondary task, coming to the conclusion that distractor-response binding effects are independent of attentional resources. Based on the results of these studies, it would be tempting to assume that binding effects, in general, are unaffected by higher central attention demands.

On the other hand, however, studies examining binding in the perceptual domain have found the binding of perceptual features to be affected by attention. Although these studies implemented different paradigms, it is worthwhile to consider the influence of attention on visual binding effects as feature binding is common to both perception and action. Treisman and Schmidt (1982) instructed participants to report digits presented on the ends of a display as their primary task, additionally, participants had to report the identity, color, and positions of the three letters presented between the two digits. Under these conditions, they observed evidence for an increased number of *illusory conjunctions*, i.e., incorrect combinations of features. Thus, they concluded that focused attention is necessary to accurately bind features. Additionally, it has also been observed that central attention is necessary for the maintenance of both bindings and individual features (e.g., Allen et al., 2012), especially for items at earlier serial positions (Hitch et al., 2020). For instance, Allen et al. (2012) found that additional central load due to a secondary task interfered with both the memory for single feature and feature bindings.

One cause for the discrepancy in the findings of visual binding and stimulus-response binding studies could lie in the methodological differences, i.e., in stimulus-response binding tasks usually only one task relevant stimulus is presented in a relatively simple display, and must be responded to immediately with a motor response, whereas in visual binding tasks, an array of stimuli are presented, which have to be held in working memory for a certain duration they are recalled from memory. Thus, such tasks explicitly require a large number of items to be held in working memory, thereby demanding more resources, and so it is perhaps not surprising that higher working memory load influences the integration and maintenance of bindings. Thus, it is possible that the load manipulations in the previous studies on stimulus-response binding did not completely drain the central attention resources, so that processes requiring very small amounts of resources were still unaffected.

The present study attempts to test this possibility by introducing a load manipulation that demands a large amount of central resources while keeping the structure and complexity of the primary task similar to previous binding studies. To this end, in both experiments, participants were instructed to carry out two tasks simultaneously, a primary task involving a prime and a probe stimulus, requiring a binary choice response and a secondary additional task consisting of a continuous working memory updating task. Binding effects were measured in the primary letter identification task. Additionally, participants performed a working memory updating task as a secondary task. The secondary task implemented has previously been shown to be effective in inducing higher cognitive load, and thereby influencing cognitive control processes [Soutschek et al., 2013; note that the present study examines a process that is more automatic than the process studied by Soutschek et al. (2013, see also section General discussion)]. Distractor-response binding effects were tested under conditions of low load, high load and no load as a control condition. The amount of load was manipulated in a block-wise manner.

Additionally, studies investigating the interaction of visual and central attention (e.g., Reimer et al., 2015; Reimer and Schubert, 2019, 2020) observed that the influence of central attention on visual attention was dependent upon the type and amount of demands placed on the attentional system. Therefore, in the present study, the specific process targeted process was also varied—maintenance vs. updating. In Experiment 1, the stimuli for the working memory updating task were presented before the prime of the choice RT task, allowing participants to update memory contents before the choice RT task began, i.e., before any event file for the RT task was formed or stimulus-response bindings were created. Thus, the memory contents were merely maintained over the duration of a single trial. In Experiment 2, the same two tasks were used; however, the working memory updating task was now presented between the integration (prime stimulus) and retrieval (probe stimulus) processes of the primary task. That is, the updating of the counters had to be carried out between integration and retrieval in the primary task instead of before the primary RT task, thus targeting a different process—updating working memory contents rather than maintaining them. In both experiments, if bindings are independent of working memory load, then there should be no difference in the binding effects between the load conditions. However, if bindings are dependent on working memory resources, then the binding effects in the high-load condition are expected to be smaller than the binding effects in the control- and low-load conditions. Additionally, a cross-experimental analysis should reveal smaller binding effects in Experiment 2 compared to Experiment 1 as in Experiment 2 the memory contents must be actively updated while maintaining an event file, whereas in Experiment 1 the memory contents must only be maintained in parallel with an event file.

## Experiment 1

The aim of Experiment 1 was to examine the effect of cognitive load on the distractor-response binding effect. To that end, a continuous updating task was presented along with the primary binding task. In Experiment 1, the secondary task was always presented in the interval before the prime of each trial to avoid any potential confounding effects of the presentation of an additional event in between the prime and the probe.

If the cognitive load does not have any effect on the distractor-response binding effect, then no difference should be observed between the binding effects in the three load conditions. In this case, a significant interaction of response relation and distractor relation, indicating a significant distractor-response binding effect, would be expected. However, no three-way interaction of response relation × distractor relation × load is expected. If, on the other hand, cognitive load influences the size of the distractor-response binding effect, then binding effects should be smaller in the high-load condition relative to the control and the low-load conditions, with the binding effect in the latter two conditions not differing in size. This would be expected to be observed in a significant three-way interaction of response relation × distractor relation × load.

*Participants*: 45 students (21 male) from the Martin-Luther University Halle-Wittenberg participated in Experiment 1 for partial course credit. The mean age of the participants was 24.30 years (range 19–31 years). All participants reported normal or corrected-to-normal eyesight. All participants provided informed consent prior to their participation in the study. The sample size of the study was calculated based on a medium-sized effect Cohen's *d* = 0.5, with an α-level of 0.05, and power of 1 – β = 0.95. The sample size calculation resulted in a sample size of 43 participants; however, as the sequence of blocks was counterbalanced across participants and a sample of 45 participants was tested.

*Design:* The experimental design consisted of a 3 (cognitive load: control vs. low load vs. high load) × 2 (response relation: repetition vs. change) × 2 (distractor relation: repetition vs. change).

*Material:* The experiment was run by using Presentation (Neurobehavioural Systems, Berkeley, CA, USA) on a standard PC attached to a 17-inch monitor with a refresh rate of 100 Hz and a standard QWERTZ keyboard. The stimuli for the primary task were the letters F and J served as targets and X and O served as distractors. Letters subtended a visual angle of 0.60° from a viewing distance of 60 cm. The responses to the primary task were made *via* a key press of the appropriate key, i.e., for the target “F” the F-key and for the target “J” the J-key. The stimuli for the secondary updating task were the mathematical operators “+” and “–” presented to the left of right of the “^*^” symbol. Responses to the secondary task were made using the number keys on the keyboard. All stimuli were presented in white on a black background.

**Figure 1:**
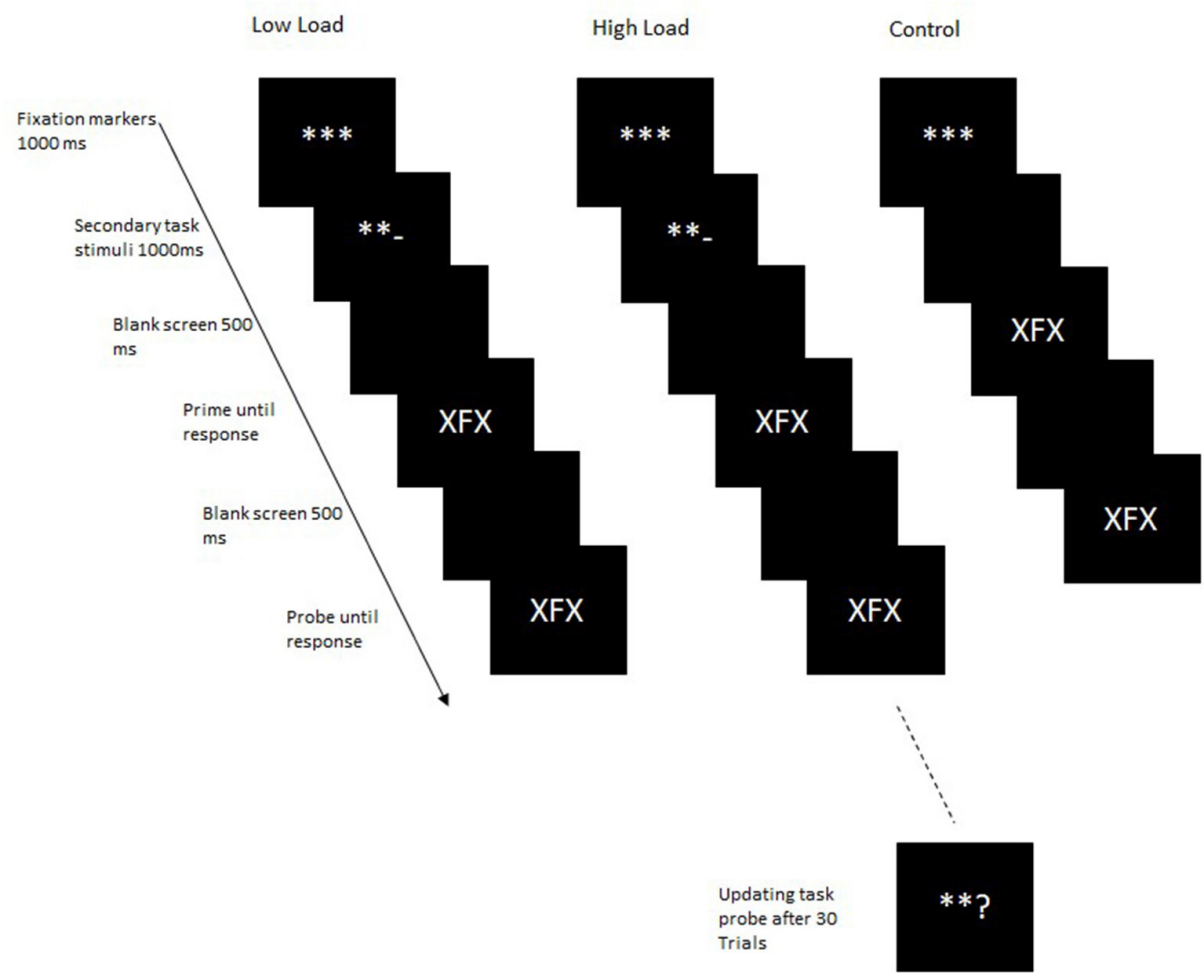
Trial structure in Experiment 1. In the control block, the secondary task stimuli were not presented.

*Procedure:* Participants were tested individually in individual chambers. Task instructions were presented on the screen. The primary task consisted of a letter identification task, in which participants were presented with a target letter flanked on either side by a flanking distractor letter (Figure 1). Targets could be either the letter “F” or the letter “J,” and distractors could be either the letter “X” or the letter “O.” Participants responded to the target “F” by pressing the F-key and the target “J” with the J-key on a standard QWERTZ keyboard.

In the primary task, participants made a speeded binary choice reaction to a centrally presented target while ignoring the flanking distractors. A factorial combination of the factors response relation (repetition vs. change) and distractor relation (repetition vs. change) resulted in four conditions repeated 48 times each within each load block. In response repetition-distractor repetition (RRDR) trials, both the response and the distractor from the prime were repeated in the probe. In response repetition-distractor change (RRDC) trials, the response from the prime was repeated in the probe but the distractor was changed. In response change-distractor repetition (RCDR) trials, a different response was required in the probe, but the distractor from the prime was repeated in the probe. In response change-distractor change (RCDC) trials, neither the response nor the distractor from the prime was repeated in the probe. Table 1 provides an example of the stimuli presented in these conditions.

**Table 1:** An example of stimuli in each of the conditions within each load block.

**Condition**	**Prime**	**Probe**
RRDR	XFX	XFX
RRDC	XFX	OFO
RCDR	XFX	XJX
RCDC	XFX	OJO

Additionally, cognitive load was varied orthogonally to the above two factors. Cognitive load was manipulated by using a continuous updating task. Participants were instructed to simultaneously maintain and update two counters, adding or subtracting in steps of two depending on the operator presented. Initially, each counter was set to a value of 50 at the beginning of the block. At the beginning of each trial, participants saw three fixation markers “^***^” at the center of the screen. One of the outer fixation markers then randomly changed to a “+” or “–” sign, indicating which counter was to be updated. The left outer fixation marker indicated one counter, and the right outer fixation marker indicated the other counter. A “+” sign indicated that two was to be added to the current value of the counter, and a “–” sign indicated that two was to be subtracted from the current value of the counter. In the secondary task, participants were instructed to maintain and update two mental counters simultaneously. Based on the presented operators, “+” or “–” participants were instructed to either add or subtract two from the current value of the counter. After 30 trials, participants were asked to enter the current value of one of the counters. As an example, if participants saw the left side marker turn to a “+” sign, they added two to the counter represented by the left marker. The value of this counter was thus 52. Participants thus maintained the values of 52 and 50 in working memory until the next trial. In the next trial, if the left side marker again turned into a “+” sign, they added two to that counter again, i.e., 54. Thus, now participants maintained the values of 54 and 50 until the next trial. In the next trial, if the right-side marker now turned into a “–” sign, they subtracted two from the counter represented by the right-side marker, i.e., 48 (thus maintaining the values of 54 and 48 until the next trial). After 30 trials, either the left- or right-side marker turned into a “?” and participants reported the current value of that counter.

Each trial began with the presentation of three fixation markers “^***^” at the center of the screen for 1,000 ms. The outer left or right fixation marker was then randomly replaced by a “+” or a “–” for 1,000 ms indicating which counter the participant was to be updated. A “+” indicated that the counter had to be updated by adding two, whereas the “–” indicated that two had to be subtracted from the value of the counter. Following a mathematical operator, a blank screen was presented for 500 ms after which the primary task was presented. The prime was presented until a response was registered. After a response to the prime was registered, a blank screen was presented for 500 ms after which the probe display was presented until a response was registered. After 30 trials, participants were asked to enter the value of either one of the secondary task counters. The response was made by typing in the value of the counter. Once the participant had made a response, they were instructed to restart both counters again from 50.

In the control condition, participants were not presented with the mathematical operators for the secondary task, instead the prime was presented after the fixation markers. In the low-load condition, participants were presented with the mathematical operators but were instructed that they were to attend to the stimuli but not to carry out the secondary task. In the high-load block, participants were presented with the mathematical operators for the secondary task and asked to perform the secondary task. Load was manipulated block-wise with the order of blocks balanced across participants, and the order of blocks varied in a Latin square. At the beginning of the experiment, participants worked through a practice block consisting of 32 trials for each of the load conditions in order to familiarize themselves with the primary task alone and with performing both tasks simultaneously. Each of the test blocks consisted of 192 trials.

*Data analyses*: The data were analyzed with IBM SPSS (Version 25). In accordance with the design, a 3 (load: high load vs. low load vs. control) × 2 (response relation: repetition vs. change) × 2 (distractor relation: repetition vs. change) multivariate ANOVA (MANOVA) with repeated measures with Pillai's trace as the criterion was conducted. Paired *t*-test was used for follow-up comparisons. A significant distractor-response binding effect is indicated by a significant interaction of response relation × distractor relation. The distractor-response binding effects can be quantified as follows: [(RRDC-RRDR) – (RCDC-RCDR)]. Larger difference scores indicate a larger binding effect. A modulating influence of load on the distractor-response binding effect would be observed in a significant three-way interaction of load × response relation × distractor relation.

### Results

*Reaction times*: Only probe RTs on trials with correct responses to the prime and the probe were analyzed (1.89% prime errors, 2.13% probe errors and 0.12% errors on prime and probe). Furthermore, RTs shorter than 200 ms (0.02%) and longer than 1.5 interquartile ranges above the third quartile as calculated for each participant (6.69%) were excluded. This resulted in an exclusion of 10.85% of the data. Three participants were excluded because they had a 0% accuracy rate in the secondary task. The mean RTs are presented in Table 2.

**Table 2:** Mean reaction times (RTs) with SD in parenthesis for Experiment 1.

	**Control**		**Low load**		**High load**	
	**Distractor repetition**	**Distractor change**	**Distractor repetition**	**Distractor change**	**Distractor repetition**	**Distractor change**
Response repetition	424 (56)	442 (59)	426 (61)	443 (64)	511 (159)	525 (165)
Response change	459 (70)	452 (70)	466 (77)	461 (81)	540 (168)	539 (170)

**Figure 2:**
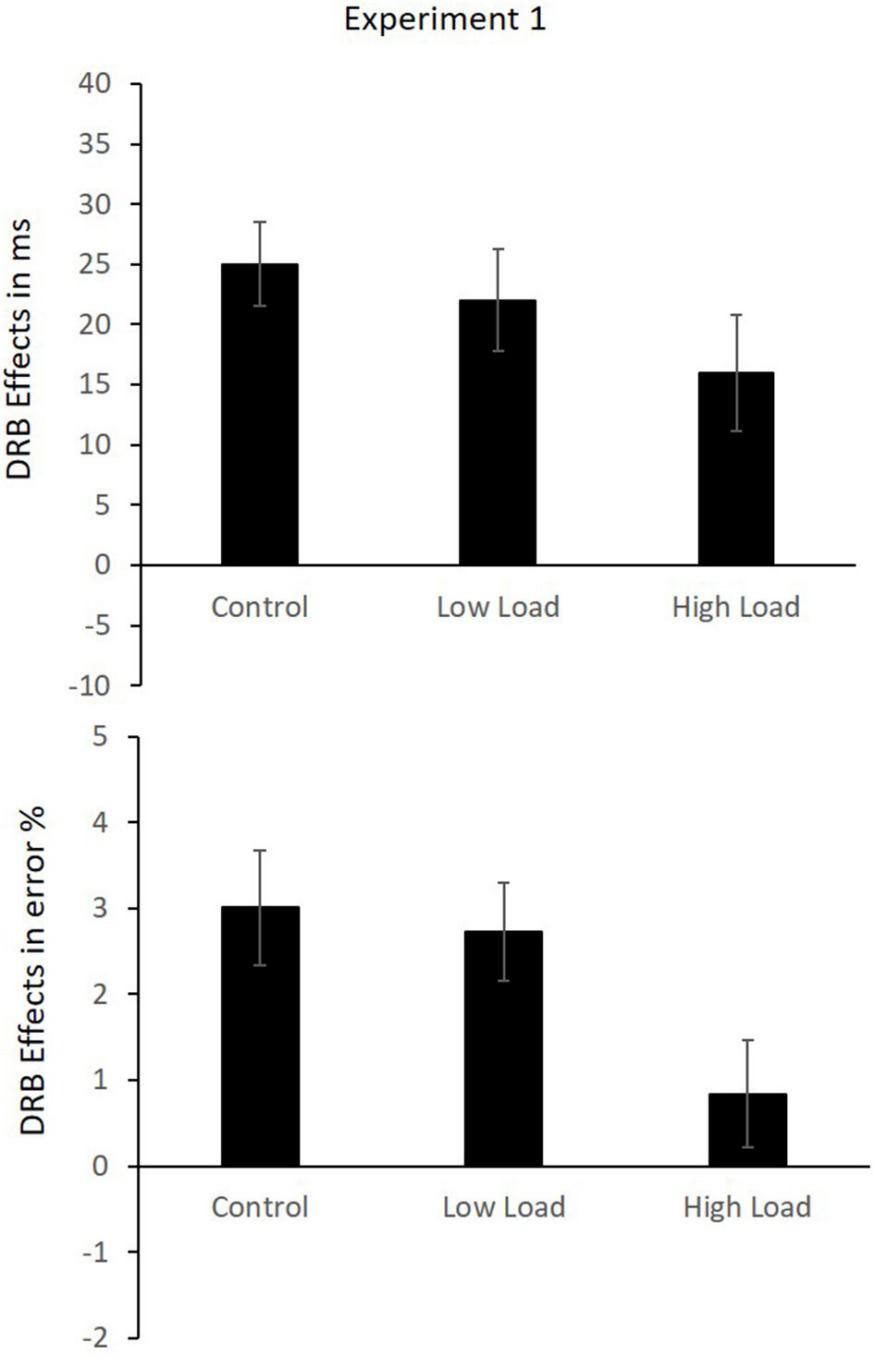
Mean DRB effects in reaction times (RTs; **Upper**) and error rates **(Lower)** in Experiment 1. Error bars indicate the SEM.

A 3 (load: high load vs. low load vs. control) × 2 (response relation: repetition vs. change) × 2 (distractor relation: repetition vs. change) MANOVA with repeated measures with Pillai's trace as the criterion was conducted. A significant main effect of load was observed, *F*_(2,40)_ = 7.87, *p* = 0.001, $\eta_{\text{p}}^{2}$ = 0.28, indicating slower responding in the high-load condition (*M* = 528 ms, SD = 164 ms) compared to the low-load (*M* = 449 ms, SD = 68 ms) condition, *t*_(41)_ = 4.01, *p* < 0.001. RTs in the high-load condition were also slower than in the control condition (*M* = 444 ms, SD = 60 ms), *t*_(41)_ = 3.96, *p* < 0.001. The low-load condition and the control condition did not differ significantly *t*_(41)_ = 0.96, *p* = 0.342. A significant main effect of response relation was observed, *F*_(1,41)_ = 21.05, *p* < 0.001, $\eta_{\text{p}}^{2}$ = 0.34, suggesting faster responding in response repetition (*M* = 460 ms, SD = 84 ms) compared to response change (*M* = 483 ms, SD = 92 ms) trials. A significant effect of distractor relation was observed, *F*_(1,41)_ = 30.32, *p* < 0.001, $\eta_{\text{p}}^{2}$ = 0.43, indicating faster responses in the distractor repetition condition (*M* = 468 ms, SD = 84 ms) compared to the distractor change condition (*M* = 475 ms, SD = 88 ms). The load × response interaction was not significant, *F*_(2,40)_ = 2.39, *p* = 0.104, $\eta_{\text{p}}^{2}$ = 0.11. The interaction of load × distractor relation was not significant, *F*_(2,40)_ = 0.12, *p* = 0.888, $\eta_{\text{p}}^{2}$ = 0.01. The interaction of response relation × distractor relation was significant, *F*_(1,41)_ = 86.63, *p* < 0.001, $\eta_{\text{p}}^{2}$ = 0.68, indicating an overall binding effect across all load conditions. Repeating the distractor while repeating the response (*M* = 451 ms, SD = 82 ms) was faster than changing a distractor while repeating the response (*M* = 469 ms, SD = 85 ms), *t*_(41)_ = 10.85, *p* < 0.001. Conversely, repeating the distractor while changing the response was slower (*M* = 485 ms, SD = 90 ms) than changing the distractor while changing the response (*M* = 481 ms, SD = 94 ms), *t*_(41)_ = 2.21, *p* = 0.033. The three-way interaction of load × response relation × distractor relation was not significant, *F*_(2,40)_ = 1.10, *p* = 0.343, $\eta_{\text{p}}^{2}$ = 0.05, indicating that the binding effects in the three load conditions did not differ; low-load *M* = 22 ms, SD = 28 ms, high-load *M* = 16 ms, SD = 31 ms and control *M* = 26 ms, SD = 23 ms (Figure 2).

*Error rates*: The same analysis was run on the error rates. The mean error rates are presented in Table 3. A significant main effect of load was observed, *F*_(2,40)_ = 23.39, *p* < 0.001, $\eta_{\text{p}}^{2}$ = 0.54, indicating lower error rates in the high-load condition (*M* = 1.47%, SD = 1.49%) compared to the low-load condition (*M* = 2.53%, SD = 2.08%), *t*_(41)_ = 5.71, *p* < 0.001. Error rates in the high-load condition were also lower than those in the control condition (*M* = 2.96%, SD = 2.43%), *t*_(41)_ = 6.45, *p* < 0.001. Error rates in the low-load condition were significantly lower than those in the control condition, *t*_(41)_ = 2.23, *p* = 0.032. A significant main effect of response relation was observed, *F*_(1,41)_ = 14.07, *p* = 0.001, $\eta_{\text{p}}^{2}$ = 0.26, indicating lower error rates in the response repetition condition (*M* = 1.69%, SD = 1.88%) compared to the response change condition (*M* = 2.95%, SD = 2.44%). The main effect of distractor relation was not significant, *F*_(1,41)_ = 0.95, *p* = 0.334, $\eta_{\text{p}}^{2}$ = 0.02. The interaction of load × response relation was significant, *F*_(2,40)_ = 6.16, *p* = 0.005, $\eta_{\text{p}}^{2}$ = 0.24, indicating that the benefit of response repetition differed across the load conditions. In the low-load condition, response repetition (*M* = 1.56%, SD = 2.19%) differed significantly from response change (*M* = 3.49, SD = 2.84%), *t*_(41)_ = 4.28, *p* < 0.001. In the high-load condition, response repetition (*M* = 1.37%, SD = 2.24%) and response change (*M* = 1.58%, SD = 1.50%) did not differ significantly, *t*_(41)_ = 0.57, *p* = 0.572. And in the control condition, response repetition (*M* = 2.13%, SD = 2.10%) differed significantly from response change (*M* = 3.79%, SD = 3.76%), *t*_(41)_ = 2.91, *p* = 0.006. The interaction of load × distractor relation was not significant, *F*_(2,40)_ = 0.13, *p* = 0.883, $\eta_{\text{p}}^{2}$ = 0.01. The interaction of response relation × distractor relation was significant, *F*_(1,41)_ = 40.58, *p* < 0.001, $\eta_{\text{p}}^{2}$ = 0.50, indicating an overall distractor-response binding effect. Repeating the distractor while repeating the response (*M* = 1.04%, SD = 1.67%) resulted in fewer errors than changing a distractor while repeating the response (*M* = 2.32%, SD = 2.38), *t*_(41)_ = 10.85, *p* < 0.001. Conversely, repeating the distractor while changing the response resulted in more errors (*M* = 3.41%, SD = 2.58%) than changing the distractor while changing the response (*M* = 2.49, SD = 2.57%), *t*_(41)_ = 2.21, *p* = 0.033. Importantly, the three-way interaction of load × response relation × distractor relation was significant, *F*_(2,40)_ = 3.58, *p* = 0.037, $\eta_{\text{p}}^{2}$ = 0.15, indicating that the distractor-response binding effect differed across the three load conditions; low-load *M* = 2.72%, SD = 3.66%, high-load *M* = 0.84%, SD = 4%, and control *M* = 3.10%, SD = 4.36% (Figure 2). We checked for a potential speed-accuracy trade-off by computing the regression slopes for the RTs and error rates on the means of all 12 conditions for each participant individually, and then tested the mean of the slopes against null in a single sample *t*-test, *t*_(40)_ = −1.75, *p* = 0.088.

**Table 3:** Mean error rates with SD in parenthesis for Experiment 1.

	**Control**		**Low load**		**High load**	
	**Distractor repetition**	**Distractor change**	**Distractor repetition**	**Distractor change**	**Distractor repetition**	**Distractor change**
Response repetition	1.28 (1.72)	2.97 (3.48)	0.73 (1.61)	2.39 (3.15)	1.13 (2.85)	1.61 (2.38)
Response change	4.45 (4.43)	3.12 (3.66)	4.02 (3.09)	2.96 (3.56)	1.76 (1.75)	1.40 (2.10)

A separate analysis of each load block was conducted to test the distractor-response binding effect within each block to pinpoint the nature of the three-way interaction of load by response relation by distractor relation. The response relation × distractor relation interaction, which indicates a distractor-response binding effect, was significant in the control block, *F*_(1,41)_ = 20.00, *p* < 0.001, $\eta_{\text{p}}^{2}$ = 0.33, and in the low-load block, *F*_(1,41)_ = 23.10, *p* < 0.001, $\eta_{\text{p}}^{2}$ = 0.36, but not in the high-load block, *F*_(1,41)_ = 1.87, *p* = 0.179, $\eta_{\text{p}}^{2}$ = 0.05, indicating that the distractor-response binding effect was present in the control and low-load blocks but not in the high-load block. Binding effects in each block are presented in Figure 2. However, a closer inspection of the effects of the load manipulation, i.e., the main effect of load, in the RTs and error rates, indicates a trend toward some sort of speed-accuracy trade-off (although, note, the speed-accuracy trade-off analyses above did not indicate a significant speed-accuracy trade-off). This makes the interpretation of the three-way interaction in the error rates somewhat complicated (see Section “Discussion”). In fact, the pattern of errors in the high-load condition mirrors a typical pattern in the binding effect, fewer errors in the RRDR condition relative to the RRDC condition and fewer errors in the RCDC condition relative to the RCDR condition. The absence of a Distractor-Response Binding (DRB) effect in this condition is thus due to the very low error rates in the condition as a whole.

### Discussion

In the first experiment, subjects were presented with a secondary task between each trial, which required them to actively update and maintain two numerical counters. In the high-load block, participants had to carry out the updating task, whereas in the low-load condition, participants were instructed to simply pay attention to the stimuli of the second task but not to carry out the updating task. In the control condition, participants were not presented with the stimuli for the updating task at all. In both the reaction times and the error rates, a significant interaction of response relation and distractor relation was observed, indicating a significant distractor-response binding effect. The crucial three-way interaction of load × response relation × distractor relation indicating that the strength of the distractor-response binding effects was modulated by load and only significant in the error rates. A closer inspection of the distractor-response binding effects within each load block indicated that there was no significant distractor-response binding effect in the error rates in the high-load block, whereas, in the control and low-load blocks, a significant distractor-response binding effect was observed. The three-way interaction in the error rates would be consistent with the assumption that the binding effect was modulated by load. However, the pattern of results is somewhat puzzling. While participants responded the slowest in the high-load condition, they also made the fewest errors in this condition. This pattern indicates that participants might have strategically focused on making fewer errors, rather than on responding fast, thus leading to the lower error rate in that condition. The interpretation of the result pattern across the RTs and error rates is thus not very straightforward. While no significant modulation of the DRB effect by load was observed in the RTs, such a modulation was indeed observed in the error rates. Nevertheless, given that the descriptive pattern across the RTs and error rates is similar, i.e., descriptively smaller DRB effects in the high-load condition, a cautious interpretation might be that even the maintenance task modulates the binding effect such that the DRB effect is attenuated in the high-load block. On the other hand, for the reasons mentioned above, caution must be exercised in such an interpretation of this interaction. Taken together, this pattern across RTs and error rates seems to be somewhat inconclusive, and does not allow a clear conclusion as to the effects of load *via* a maintenance task on DRB effects. In Experiment 2, the effect of load on DRB effects was tested by using a different task.

## Experiment 2

**Figure 3:**
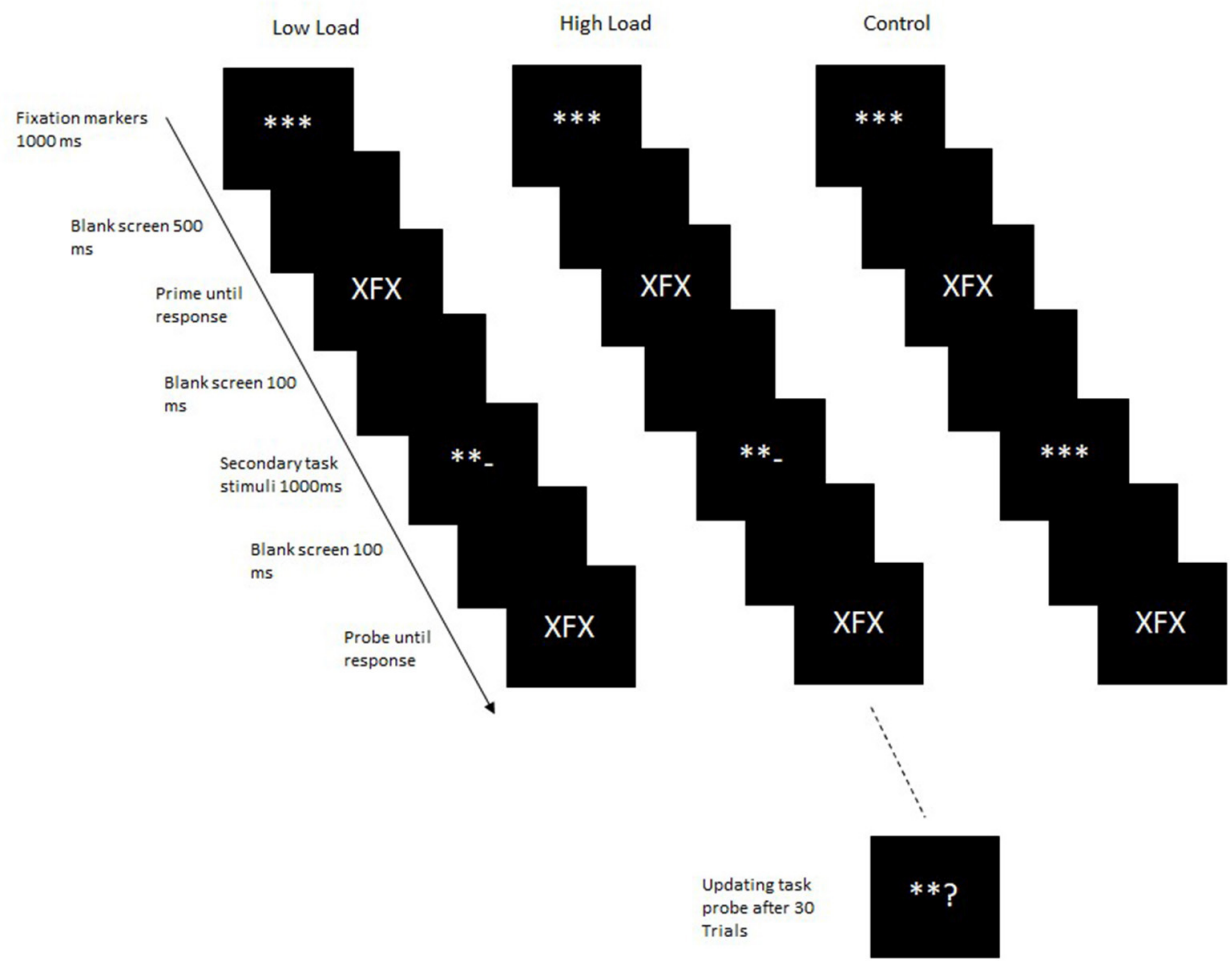
Trial structure in Experiment 2. In the control block, the secondary task stimuli were not presented. Instead, fixation markers were presented for the duration.

Based on the findings that the interaction of central attention and visual attention depends upon the type of task and the specific process being targeted (Reimer et al., 2015; Reimer and Schubert, 2019, 2020), a different process was targeted in the second experiment. Thus, in the second experiment, the effect of updating a secondary task between the integration and retrieval processes of a binding task was examined. In the first experiment, the stimuli for the updating task were always updated before the integration of the event file and its subsequent retrieval. Thus, although participants had to maintain the updated secondary task during the integration and retrieval phases, they did not have to actively update working memory contents within the primary task. In the second experiment, the updated task stimuli were presented between the prime and the probe so that working memory contents had to be updated after the event file was integrated and before it was retrieved. Thus, in this case, a different processes is targeted; updating of working memory contents instead of maintenance. In most binding tasks, the retrieval follows the integration process directly, with no intervening stimuli (however, see Frings and Rothermund, 2011; Hommel and Frings, 2020; see section General Discussion). A few studies specifically examining the influence of intervening stimuli on stimulus-response binding effects (e.g., Hommel and Frings, 2020) do not show any disruption of binding effects due to intervening stimuli. Therefore, we expected that the mere presentation of the updating task stimuli as intervening stimuli would not affect bindings. The aim of the second experiment was to examine the influence of active updating of memory contents on binding processes. In Experiment 2, as in Experiment 1, participants had to update working memory contents in the secondary task in the high-load condition. In the low-load condition, as in Experiment 1, they were instructed to only attend to the secondary task stimuli but not to carry out the task. In the control condition, the secondary task stimuli were not presented, rather fixation markers were presented for the same duration as the secondary task stimuli in the high- and low-load conditions (Figure 3). Distractor-response binding should be observed in the control block as here no secondary task stimuli are presented, and thus memory contents do not have to be updated. Additionally, as no intervening stimuli are presented, this condition most closely resembles the standard binding paradigm. If distractor-response binding effects are not modulated by working memory updating processes, then significant binding effects should be observed in all load conditions. On the other hand, if bindings are disrupted by updating processes, then there should be no significant binding effect in the high-load condition. If the type of load manipulation, maintenance of memory contents and bindings versus updating of memory contents while maintaining bindings, have different effects on distractor-response binding effects then this difference should be evident in a cross experiment analyses. As the updating of memory content while maintaining bindings is arguably a more resource-intensive operation, we expect the binding effects in Experiment 2 to be significantly smaller than those in Experiment 1.

*Participants*: 45 students (15 male) from the Martin-Luther University Halle-Wittenberg participated in Experiment 1 for partial course credit. The mean age of the participants was 22.20 years (range 18–63 years). All participants reported normal or corrected-to-normal eyesight. All participants provided informed consent prior to their participation in the study. The sample size of the study was calculated as for Experiment 1.

*Design, materials and procedure*: The procedure of Experiment 2 was similar to that of Experiment 1 with the following exceptions; in the high-load and low-load blocks, the mathematical operators for the secondary task were presented after the prime for 1,000 ms^1^ and was then followed by the probe, whereas in the control block fixation markers were presented for 800 ms. Before and after the secondary task stimuli or fixation markers, a blank screen was displayed for 100 ms. Apart from these changes, everything else remained the same as in Experiment 1. The data analyses were performed similar to Experiment 1.

*Reaction times*: Only probe RTs on trials with correct responses to the prime and the probe were analyzed (1.44% prime errors, 2.35% probe errors, and 0.06% errors on both prime and probe). Furthermore, RTs shorter than 200 ms (0.004%) and longer than 1.5 interquartile ranges above the third quartile, as calculated for each participant (9.34%) were excluded. This resulted in a rejection of 13.20% of the data. Additionally, three participants were excluded because they had a 0% accuracy rate in the secondary task. The mean RTs are presented in Table 4.

**Table 4:** Mean RTs with SD in parenthesis for Experiment 2.

	**Control**		**Low load**		**High load**	
	**Distractor repetition**	**Distractor change**	**Distractor repetition**	**Distractor change**	**Distractor repetition**	**Distractor change**
Response repetition	495 (84)	506 (83)	571 (235)	558 (206)	885 (428)	887 (448)
Response change	527 (90)	525 (88)	579 (214)	564 (179)	878 (421)	865 (412)

A 3 (load: high load vs. low load vs. control) × 2 (response relation: repetition vs. change) × 2 (distractor relation: repetition vs. change) MANOVA with repeated measures was conducted with Pillai's trace as the criterion. A significant main effect of load was observed, *F*_(2,40)_ = 19.16, *p* < 0.001, $\eta_{\text{p}}^{2}$ = 0.49, RTs in the high-load condition (*M* = 879 ms, SD = 425 ms) were longer than RTs in the low-load condition (*M* = 568 ms, SD = 206 ms), *t*_(41)_ = 5.48, *p* < 0.001, and longer than the control condition (*M* = 513 ms, SD = 85 ms), *t*_(41)_ = 6.25, *p* < 0.001. The difference between RTs in low-load condition and control condition missed the significance, *t*_(41)_ = 2.01, *p* = 0.051. The main effect of response relation was not significant, *F*_(1,41)_ = 1.91, *p* = 0.175, $\eta_{\text{p}}^{2}$ = 0.04. The main effect of distractor relation was not significant, *F*_(1,41)_ = 1.39, *p* = 0.245, $\eta_{\text{p}}^{2}$ = 0.03. The load × response interaction was significant, *F*_(2,40)_ = 12.16, *p* < 0.001, $\eta_{\text{p}}^{2}$ = 0.38, indicating that the benefit of response repetition vs. response change differed in magnitude across the load conditions. In the low-load condition, response repetition (*M* = 565 ms, SD = 218 ms) and response change (*M* = 571 ms, SD = 196 ms) did not differ significantly, *t*_(41)_ = 1.12, *p* = 0.268. In the high-load condition as well response repetition (*M* = 886 ms, SD = 436 ms) and response change (*M* = 872 ms, SD = 416 ms), conditions did not differ significantly, *t*_(41)_ = 1.34, *p* = 0.170. Only in the control condition, there was a significant difference observed in response repetition (*M* = 500 ms, SD = 83 ms) and response change (*M* = 526 ms, SD = 88 ms), *t*_(41)_ = 7.57, *p* < 0.001. The interaction of load × distractor relation was not significant, *F*_(2,40)_ = 2.97, *p* = 0.062, $\eta_{\text{p}}^{2}$ = 0.13. The interaction of response relation × distractor relation was not significant, *F*_(1,41)_ = 1.61, *p* = 0.211, $\eta_{\text{p}}^{2}$ = 0.04, indicating the absence of an overall distractor-response binding effect across all load conditions. The three-way interaction of load × response relation × distractor relation was not significant, *F*_(2,40)_ = 0.47, *p* = 0.628, $\eta_{\text{p}}^{2}$ = 0.02. A control analysis with block sequence as a factor indicated that sequence did not interact either with the binding effect, response relation × distractor relation × block sequence, *F*_(2,38)_ = 0.95, *p* = 0.396, $\eta_{\text{p}}^{2}$ = 0.05, nor did it modulate the three-way interaction, load × response relation × distractor relation × block sequence, *F*_(4,78)_ = 1.58, *p* = 0.183, $\eta_{\text{p}}^{2}$ = 0.08.

**Figure 4:**
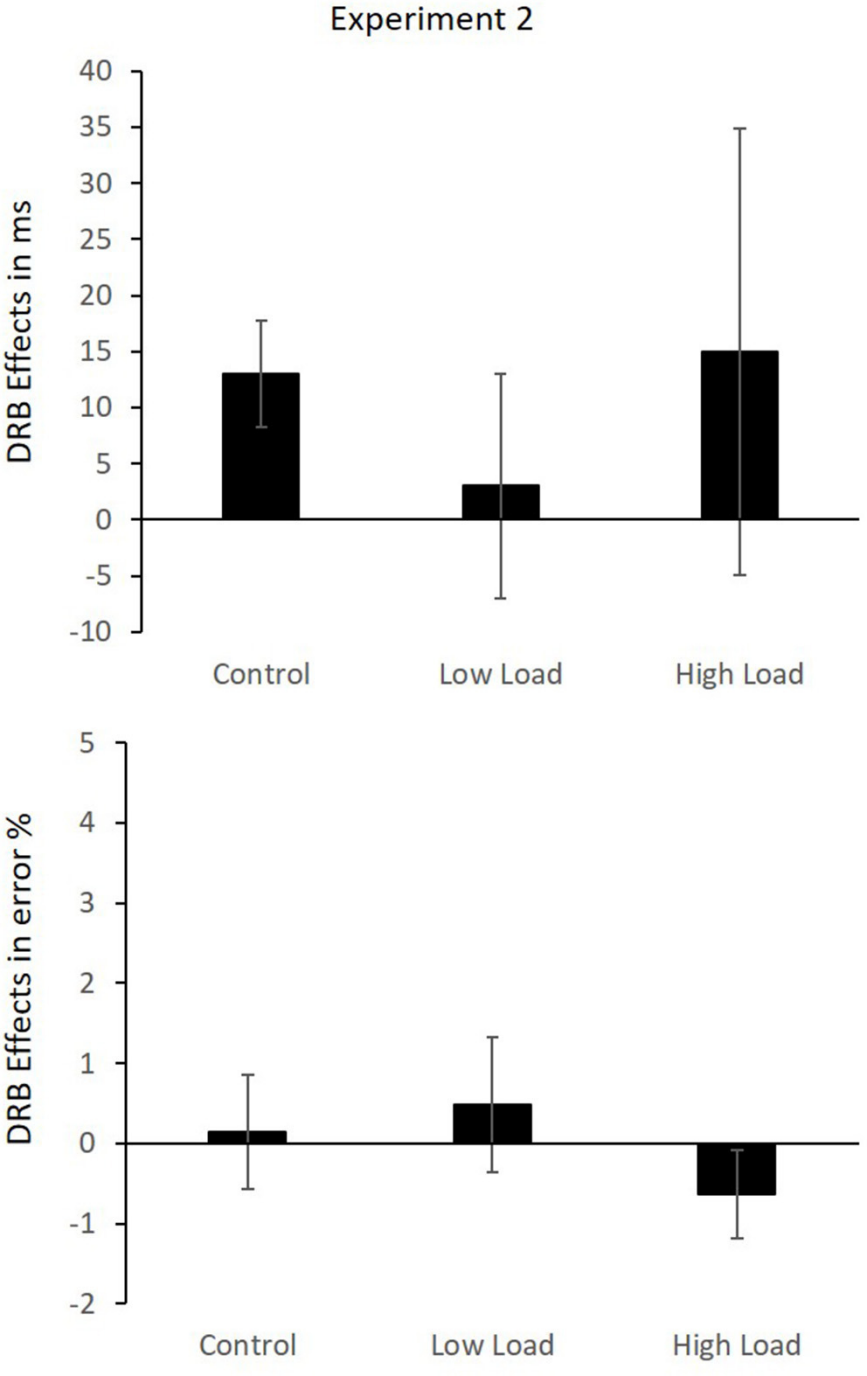
Mean DRB effects in RTs **(Upper)** and error rates **(Lower)** in Experiment 2. Error bars indicate the SEM.

The results indicate no binding effect over all load conditions and no modulation due to load. However, as we predicted a significant binding effect at least in the control conditions and this condition most closely resembles the standard binding paradigm, we conducted a *post-hoc* analysis of the binding effects separately for three load blocks separately. It showed that the response relation × distractor relation interaction, which is indicative of a distractor-response binding effect, was significant in the control block, *F*_(1,41)_ = 7.08, *p* = 0.011, $\eta_{\text{p}}^{2}$ = 0.15, whereas it was not significant in the low load and high load blocks, *F*_(1,41)_ = 0.11, *p* = 0.742, $\eta_{\text{p}}^{2}$ = 0.00, and *F*_(1,41)_ = 0.64, *p* = 0.430, $\eta_{\text{p}}^{2}$ = 0.02, respectively (Figure 4).

*Error rates*: The same analysis was run on the error rates. Only trials with correct responses to the prime were considered in the analysis. The mean error rates are presented in Table 5. The main effect of load was not significant, *F*_(2,40)_ = 0.49, *p* = 0.615, $\eta_{\text{p}}^{2}$ = 0.02. The main effect of response relation was not significant, *F*_(1,41)_ = 2.28, *p* = 0.138, $\eta_{\text{p}}^{2}$ = 0.05. The main effect of distractor relation was not significant, *F*_(1,41)_ = 0.27, *p* = 0.869, $\eta_{\text{p}}^{2}$ = 0.00. The interaction of load × response relation was significant, *F*_(2,40)_ = 12.62, *p* < 0.001, $\eta_{\text{p}}^{2}$ = 0.39, indicating that the benefit of response repetition differed across the load conditions. In the low-load condition, the difference between response repetition (*M* = 2.40%, SD = 1.89%) and response change (*M* = 2.70%, SD = 2.62%) was not significant, *t*_(41)_ = 0.83, *p* = 0.413. Similarly, in the high-load condition, the difference between response repetition (*M* = 2.65%, SD = 2.15%) and response change (*M* = 1.96%, SD = 1.97%) was not significantly different, *t*_(41)_ = 1.82, *p* = 0.076. Only in the control condition, the difference between response repetition (*M* = 1.56%, SD = 1.42%) vs. response change (*M* = 3.21%, SD = 3.35%) was significantly different, *t*_(41)_ = 4.06, *p* < 0.001. The interaction of load × distractor relation was not significant, *F*_(2,40)_ = 0.91 *p* = 0.410, $\eta_{\text{p}}^{2}$ = 0.04. The interaction of response relation × distractor relation was not significant, *F*_(1,41)_ = 0.00, *p* = 0.989, $\eta_{\text{p}}^{2}$ = 0.00, indicating the absence of an overall distractor-response binding effect. The three-way interaction of load × response relation × distractor relation was also not significant, *F*_(2,40)_ = 0.91, *p* = 0.412, $\eta_{\text{p}}^{2}$ = 0.04. A control analysis with block sequence as a factor indicated that sequence did not interact either with the binding effect, response relation × distractor relation × block sequence, *F*_(4,38)_ = 0.15, *p* = 0.863, $\eta_{\text{p}}^{2}$ = 0.01, nor did it further modulate load by binding interaction, load × response relation × distractor relation × block sequence, *F*_(4,78)_ = 2.23, *p* = 0.074, $\eta_{\text{p}}^{2}$ = 0.10.

**Table 5:** Mean error rates with SD in parenthesis for Experiment 2.

	**Control**		**Low load**		**High load**	
	**Distractor repetition**	**Distractor change**	**Distractor repetition**	**Distractor change**	**Distractor repetition**	**Distractor change**
Response repetition	1.38 (1.75)	1.73 (2.03)	2.31 (2.72)	2.49 (2.22)	2.98 (2.52)	2.34 (2.77)
Response change	3.09 (3.91)	3.31 (3.80)	2.84 (3.35)	2.54 (3.00)	1.96 (2.35)	1.95 (2.25)

As with the RTs, an analysis of three load blocks independently was conducted. The response relation × distractor relation interaction was not significant in any of the three, low-load *F*_(1,41)_ = 0.32, *p* = 0.575, $\eta_{\text{p}}^{2}$ = 0.01, high-load block, *F*_(1,41)_ = 1.31, *p* = 0.260, $\eta_{\text{p}}^{2}$ = 0.03, and control block, *F*_(1,41)_ = 0.04, *p* = 0.847, $\eta_{\text{p}}^{2}$ = 0.00. Binding effects in each block are presented in Figure 4.

### Discussion

In Experiment 2, the secondary task had to be actively updated between the integration and retrieval processes of the primary task. Specifically, the counters had to be updated between the prime and the probe. The results indicate that updating of working memory contents resulted in a disruption of distractor-response bindings. No significant response relation × distractor relation interaction was observed indicating that there was no distractor-response binding effect. Additionally, the three-way interaction of response relation × distractor relation × load was not significant. A *post-hoc* analysis of each block separately indicated that although no binding effect was observed in the low- and high-load block, a significant binding effect was observed in the control block. This finding conforms to the hypothesis that a significant binding effect should be observed in the control block as this condition includes neither any cognitive load, i.e., updating of working memory contents nor any intervening stimuli were presented. However, the absence of a significant binding effect in the low-load condition is contrary to the predictions. Based on the previous findings regarding intervening stimuli, significant binding effects would be expected in this condition as participants only had to attend to the intervening stimuli but not to carry out the secondary task. This finding makes the interpretation of the results of Experiment 2 puzzling as it would indicate that an intervening stimulus does, in fact, disrupt bindings. One possible explanation for the absence of binding effects in this condition could be that participants still carried out the memory updating task as in the low-load condition even though they did not have to. However, this assumption is purely speculative, and indeed, an examination of the main effect of load on RTs indicates a non-significant difference in low-load and control blocks (note however, *p* = 0.051). At the same time, an examination of the effect of load on RTs indicates a significant difference between the high- and low-load block (*p* < 0.001), indicating a lower load in the low-load block. Thus, the lack of binding effects in the low-load block cannot be accounted for only in terms of load, i.e., if as mentioned above participants carried out the updating task even in the low-load blocks.

*Cross-experimental analysis*: In order to test whether presenting the secondary task in between the prime and the probe significantly modulated the distractor-response binding effect relative to presenting the secondary task before the prime, a cross-experimental analysis was conducted on the computed distractor-response binding effects in each load block in each experiment. A mixed MANOVA with Pillai's trace was computed with the factor load (high load vs. low load vs. control) as a within-subject factor and experiment (Experiment 1 vs. 2) as a between-subject factor. In the RTs, descriptively the binding effects in all three load conditions were very similar, the control condition (*M* = 19 ms, SD = 28 ms), low-load condition (*M* = 13 ms, SD = 50 ms) and finally the high-load condition (*M* = 16 ms, SD = 93 ms). This pattern was reflected in the inferential statistics, none of the main effects nor the interaction was significant, all *F*s < 1.55 and all *p*s > 0.217. In the error rates, descriptive pattern indicated similar binding effects for the control (*M* = 1.57%, SD = 4.69%) and low-load condition (*M* = 1.60%, SD = 4.76%), and a smaller binding effect in the high-load condition (*M* = 0.11%, SD = 3.85%). This pattern was confirmed by a significant main effect of load, *F*_(2,81)_ = 4.10, *p* = 0.020, $\eta_{\text{p}}^{2}$ = 0.09. The main effect of experiment was also significant, *F*_(1,82)_ = 17.25, *p* < 0.001, $\eta_{\text{p}}^{2}$ = 0.17, indicating larger binding effects in Experiment 1 compared to Experiment 2. The interaction of experiment × load was not significant, *F*_(2,82)_ = 0.57, *p* = 0.568, $\eta_{\text{p}}^{2}$ = 0.01.

The relevant finding of the cross-experimental comparison relates to the main effect of experiment. A significant main effect of experiment indicates that the binding effects in Experiment 1 were larger than the binding effects in Experiment 2, confirming the hypothesis that updating working memory contents while maintaining the event file leads to smaller binding effects than when memory content must merely be maintained in parallel with an event file, as in Experiment 1.

## General Discussion

In two experiments, the influence of two different types of cognitive load manipulations targeting different processes on distractor-response binding effects was examined. To this end, participants carried out a continuous updating task together with the primary task. In Experiment 1, the secondary task stimuli were presented at the beginning of each trial of the primary task, thus the memory content was updated before participants responded to the prime and the probe of the primary task, and the memory content had to be maintained over the duration of the trial along with the event file/stimulus-response bindings. In Experiment 2, the secondary task stimuli were presented between the prime and the probe, thus requiring an update of the working memory contents while an event file had to simultaneously be maintained. In both experiments, if cognitive load influences binding effects, smaller binding effects were predicted in the high-load conditions relative to the low-load and control conditions, and the latter two conditions were not expected to be significantly different. In Experiment 2, actively updating memory contents in the secondary task while maintaining stimulus-response bindings was assumed to require more resources, and therefore smaller binding effects were expected in Experiment 2 relative to Experiment 1.

The observed results lend support to the predictions with respect to the effect of cognitive load on stimulus-response bindings to different degrees; in Experiment 1 no reliable differences were observed in the binding effects in the three load conditions, suggesting that even with more demanding tasks, merely maintaining contents in working memory does not necessarily disrupt binding effects. However, the results of Experiment 2 largely conformed to the predictions of an influence of cognitive load on binding effects. No significant binding effects were observed in the low-load and high-load blocks, whereas a significant binding effect was observed in the control block. Only the prediction for the low-load block was not confirmed. Based on the previous findings with regard to intervening stimuli, a significant binding effect would be expected in the low-load condition. Importantly, however, the cross-experimental analysis indicated smaller binding effects in Experiment 2 relative to Experiment 1, suggesting that the disruption of event files or bindings is dependent upon the type of load manipulation, i.e., the specific process being affected. If memory contents must only be maintained in parallel with bindings, binding effects are observed. However, if memory contents must be actively updated while an event file is maintained, then bindings are disrupted.

### Binding and Cognitive Load

Binding theories differ on whether attention is required for successful binding of features. Treisman's FIT of attention, an attention theory, suggested that attention was necessary to efficiently bind stimulus features. The theory of event coding (Hommel et al., 2001; Hommel, 2004), on the other hand, does not explicitly assume attentional resources to be necessary for feature binding, rather it refers to *intentional* processes, i.e., weighting of features based on their relevance to the action goals (Memelink and Hommel, 2013). Features with higher weights are more likely to be included in event files or bindings. Thus, both binding theories have differing views on the influence of central attention on binding effects. Both also gave rise to studies confirming the respective views (e.g., Treisman and Schmidt, 1982; Hommel, 2005).

The results of Experiment 1 did not allow for a clear conclusion; however, a cautious interpretation of the data pattern might be that the binding effects were not reliably different across the three load conditions. This is in line with the previous studies examining the effects of central load on stimulus-response bindings. It is interesting to note that the secondary task implemented in the current studies has been observed to reliably modulate cognitive control (e.g., post-conflict adaptation) with tasks such as the Stroop task (Soutschek et al., 2013). The results of Experiment 1 would seem to imply that simply maintaining items in working memory does not reliably interfere with stimulus-response binding effects. In Experiment 2, as the secondary task had to be actively updated while maintaining the event file, we hypothesized that this further increased demands on central attention, and should result in smaller binding effects in Experiment 2 relative to Experiment 1. The cross-experimental analysis indicated that this is indeed the case, binding effects in Experiment 2 are smaller than binding effects in Experiment 1. In fact, in Experiment 2 binding effects were only significant in the control condition, whereas in Experiment 1, binding effects were observed in all conditions. the results of Experiment 1 and the cross-experimental analysis taken together are in accordance with the hypothesis that cognitive load does modulate stimulus-response binding effects if the load manipulation targets the relevant processes. A high load can lead to smaller binding effects compared to the conditions with lower load, especially, when the items in working memory must be actively updated while also maintaining the bindings. The findings indicate that the interaction of visual and central attention is not necessarily straightforward, rather it is dependent upon the type of tasks involved. Analogous findings were also reported in a different paradigm by Reimer and Schubert; Reimer and Schubert (2019; 2020; Reimer et al., 2015, see below).

It is interesting to note that two different theories on feature binding come to different conclusions regarding the influence of central attention on feature binding. One cause for the differences in the observed influence of attention on binding effects could be different systems for visual–visual feature binding and visual–motor feature bindings. For instance, it has been observed that certain manipulations affect only visual–visual feature binding without modulating visuo–motor binding, e.g., caffeine (Colzato et al., 2005) and alcohol (Colzato et al., 2004). A further possibility, as mentioned previously, could lie in the methods implemented to investigate binding effects. Specifically, the difference in primary task demands might also explain the differing findings with respect to the influence of cognitive load on visual–visual and visuo–motor bindings. Thus, it is possible that visual–visual bindings are more susceptible to attentional demands than visuo–motor bindings.

The results of the cross-experimental analysis indicate a further possibility for the differences; these disruptions are only observed if the relevant process is targeted. The results of the present study indicate that, under sufficiently high cognitive load, stimulus-response bindings are reduced, i.e., when participants must actively update working memory contents but not while maintaining working memory contents. This pattern across both studies is in line with the findings of the studies that have examined the interaction of visual attention and central attention (e.g., Reimer et al., 2015; Reimer and Schubert, 2019). Reimer et al. (2015); Reimer and Schubert (2019) implemented a dual task paradigm with overlapping tasks and found that visual search processes could operate even when the response selection stage of the choice RT task was active. The authors thus concluded that visual attention is not subject to central capacity limitations. However, an increase in the complexity of Task 2 (Reimer and Schubert, 2020) by, for instance, implementing a triple conjunction search resulted in a different pattern of results. In this case, there was no evidence that visual search processes were operating during the response selection stage of the choice RT task, thus indicating that with sufficiently complex search tasks, visual attention is subject to central capacity limitations. In the present experiments, an analogous pattern of results is observed. Although the present result pattern suggests that updating working memory contents disrupt binding effects, the specific mechanism (i.e., integration, maintenance, or retrieval) that is disrupted cannot be specified with the present experimental design. Based on the findings of Allen et al. (2012), one could speculate that the integration is not disrupted, rather either the maintenance or retrieval process is disrupted. However, further research is required to specify exactly which of these mechanisms are affected.

The present findings indicate further a boundary condition for stimulus-response bindings. Even though stimulus-response bindings are automatic, they are still subject to certain boundary conditions. For instance, Hommel (1998) found that stimulus-response bindings are more reliable for stimulus features, which are relevant to the present task or action goals. Memelink and Hommel (2013) referred to this as intentional weighting, i.e., the weights of the individual features are adjusted as per the intentions of the actor, and the higher the weights, the more likely that the feature will be bound to other features. Similarly, Singh et al. (2018) found that directing attention to one of two irrelevant stimulus features *via* a secondary task resulted in larger binding effects for that particular feature. Dreisbach and Haider (2009) found that distractor-response binding effects completely disappeared if participants were instructed with a task set, which required them to focus on specific features of the stimuli rather than on specific stimulus-response mappings. Thus, attentional allocation can modulate stimulus-response binding effects, reducing the magnitude of the effects when not enough attention was allocated to the stimuli or features. The present study adds to this body of evidence by showing that not only such feature-specific attention allocation or weighting can influence stimulus-response binding effects, rather even demands on central attentional resources modulate binding effects and reduce the strength of the binding effects under sufficiently large demands on central attentional resources.

Furthermore, the present results also hint at the location of the storage of bindings, i.e., working memory. As an increase in working memory load in an updating task results in a smaller binding effect, it is plausible to assume that these bindings might be stored in working memory. A few studies on visual–visual binding paradigms come to similar conclusions regarding the storage of bindings in the visual working memory (e.g., Allen et al., 2006, 2012). The issue of where stimulus-response bindings might be stored has not yet received too much attention, and the present study might be interpreted as pointing toward the working memory as a possible storage location (see also Schubert and Strobach, 2018; Oberauer, 2019; Kübler et al., 2021).

An additional interesting finding relates to the influence of intervening stimuli on stimulus-response bindings. In Experiment 2, the stimuli for the secondary task were presented between the prime and the probe of the primary task. A few studies examining the influence of intervening stimuli on stimulus-response bindings generally find that the mere presentation of a stimulus between the prime and probe does not interfere with bindings (e.g., Frings and Rothermund, 2011; Hommel and Frings, 2020). The absence of binding effects in the low-load condition of the present Experiment 2 is unexpected. Significant binding effects would be expected in this condition as the intervening stimuli do not have to be processed, i.e., the updating task did not have to be carried out. Thus, this condition is similar to previous studies, in which the intervening stimuli could be ignored. However, no significant binding effects were observed in this block in the present study. There are two possible reasons for this; firstly, intervening stimuli do interfere with stimulus-response bindings under certain situations. Evidence supporting this assumption comes from a comparison of the effects of load in Experiment 2. Statistically, the load effects in the low-load and control condition did not differ (however, given the value of *p* is 0.051 it could be argued that this difference is close to being significant). Thus, load (alone) is unlikely account for the absence of binding effects in the low-load condition. This might indicate that intervening stimuli, in certain situations, might disrupt binding processes. Additionally, although perceptual load was not directly manipulated in this study, it could be argued that the intervening stimuli increased the perceptual load in Experiment 2. According to Lavie's (2005) perceptual load theory, increased perceptual load leads to smaller distractor interference effects, thus this might explain the findings in the low-load condition. A further possible explanation that cannot be ruled out is that participants might have carried out the updating task to a certain extent in the low-load block as well, thus disrupting binding effects.

In conclusion, the present study provides evidence that, along with factors, like task relevance and attentional set, spatial and feature-based attentional allocation, even increased cognitive load can affect stimulus-response binding effects. Specifically, while maintenance of a higher load does not seem to reliably disrupt stimulus-response binding effects, updating of working memory does. Additionally, a further more tentative interpretation is in regard to working memory as the storage location of stimulus-response bindings.

## Data Availability Statement

The raw data supporting the conclusions of this article will be made available by the authors, without undue reservation.

## Ethics Statement

The studies involving human participants were reviewed and approved by Ethics Commission of the DGPs (German Psychological Society). The patients/participants provided their written informed consent to participate in this study.

## Author Contributions

Both authors contributed to the design of the study, TSi ran the study, analyzed the data, and wrote the first draft, both authors contributed to the manuscript revisions.

## Conflict of Interest

The authors declare that the research was conducted in the absence of any commercial or financial relationships that could be construed as a potential conflict of interest.

## Publisher's Note

All claims expressed in this article are solely those of the authors and do not necessarily represent those of their affiliated organizations, or those of the publisher, the editors and the reviewers. Any product that may be evaluated in this article, or claim that may be made by its manufacturer, is not guaranteed or endorsed by the publisher.

## Footnotes

^1^In one order sequence, the secondary task stimuli were only presented for 800 ms; however, an analysis with block sequence as a factor did not reveal a significant interaction with the factor block sequence.
